# Improving benchmarking by using an explicit framework for the development of composite indicators: an example using pediatric quality of care

**DOI:** 10.1186/1748-5908-5-13

**Published:** 2010-02-09

**Authors:** Jochen Profit, Katri V Typpo, Sylvia J Hysong, LeChauncy D Woodard, Michael A Kallen, Laura A Petersen

**Affiliations:** 1 Department of Pediatrics, Baylor College of Medicine, Texas Children's Hospital, Houston, TX, USA; 2 Section of Health Services Research, Department of Medicine, Baylor College of Medicine, Houston, TX, USA; 3 Houston Veterans Affairs (VA) Health Services Research and Development Center of Excellence, Michael E. DeBakey VA Medical Center, Houston, TX, USA; 4 University of Arizona Health Sciences Center, Department of Pediatrics, Section of Pediatric Critical Care Medicine, Tucson, AZ, USA; 5 The University of Texas M. D. Anderson Cancer Center, Department of General Internal Medicine, Ambulatory Treatment and Emergency Care, Houston, TX, USA

## Abstract

**Background:**

The measurement of healthcare provider performance is becoming more widespread. Physicians have been guarded about performance measurement, in part because the methodology for comparative measurement of care quality is underdeveloped. Comprehensive quality improvement will require comprehensive measurement, implying the aggregation of multiple quality metrics into composite indicators.

**Objective:**

To present a conceptual framework to develop comprehensive, robust, and transparent composite indicators of pediatric care quality, and to highlight aspects specific to quality measurement in children.

**Methods:**

We reviewed the scientific literature on composite indicator development, health systems, and quality measurement in the pediatric healthcare setting. Frameworks were selected for explicitness and applicability to a hospital-based measurement system.

**Results:**

We synthesized various frameworks into a comprehensive model for the development of composite indicators of quality of care. Among its key premises, the model proposes identifying structural, process, and outcome metrics for each of the Institute of Medicine's six domains of quality (safety, effectiveness, efficiency, patient-centeredness, timeliness, and equity) and presents a step-by-step framework for embedding the quality of care measurement model into composite indicator development.

**Conclusions:**

The framework presented offers researchers an explicit path to composite indicator development. Without a scientifically robust and comprehensive approach to measurement of the quality of healthcare, performance measurement will ultimately fail to achieve its quality improvement goals.

## Background

In recent years, composite indicators of care quality have been used more widely to measure and track provider performance in adult medicine [1-7]. In pediatrics, interest in provider healthcare performance is rising. Various countries, such as the United Kingdom, Canada, and Australia, are developing scorecards that include measures of pediatric healthcare quality [8-10]. Resources for healthcare are finite, and high-income countries are facing rising pressures to maximize the value of healthcare expenditures. Information on provider performance can reduce the information deficit between purchasers and providers of healthcare, providing incentives for purchasers and consumers of services to use the best providers, and for providers to improve performance. Composite indicators in healthcare thus have come into wider use largely as a by-product of so called 'value-based purchasing' initiatives, where payers reimburse providers based on comparative performance (benchmarking) [11-13].

Composite indicators can provide global insights and trends about quality not just for external benchmarking against other providers or institutions, but also facilitate quality improvement efforts within institutions by identifying areas of healthcare quality that need improvement. While composite indicators may be a useful addition to the quality improvement toolbox, their development is complex, and the editorial choices required of developers may significantly influence performance ratings [14]. Therefore, development must be explicit and transparent.

The unique contribution and purpose of this paper is to advocate for using composite indicators as an approach to measure quality in pediatrics, and to present a framework for the development of composite indicators based on a combination of previously presented frameworks on both quality measurement and composite indicator development. The final approach to composite indicator development is the result of a combination of approaches described by Profit and colleagues with methods developed by the European Commission Joint Research Center (EC-JRC) and the Organization for Economic Cooperation and Development (OECD), henceforth simplified as JRC [12,15]. In the Discussion section, we will spotlight pediatric-specific aspects in composite indicator development that require empirical research. These include paucity of interactions with the healthcare system, paucity of critical health outcomes, and availability of quality of life and prevention metrics. We will focus on aspects important to pediatrics because aggregate performance measurement is comparatively new to this field. However, we believe that the application of this conceptual framework provides a comprehensive roadmap for the continuous improvement of quality measurement for all populations.

### Composite indicators of quality

Composite indicators of quality combine multiple metrics of quality into an aggregate score. Table 1 (adapted from Nardo [15]) summarizes the advantages and disadvantages of using composite indicators, regardless of field or purpose. We will discuss the advantages and disadvantages of composite indicators focusing on their two probable uses, benchmarking and quality improvement.

**Table 1:** Advantages and disadvantages of composite indicators

Advantage	Disadvantage
• Facilitate communication with other stakeholders and promote accountability • Summarize complex issues for decision-makers • Facilitate benchmarking • Assess progress over time • Induce innovation in quality improvement • Encourage system-based improvement	• Provide misleading messages about quality if poorly constructed or misinterpreted • Lead to simplistic policy conclusions • Can be misused, if the construction process is not transparent and lacks sound statistical or conceptual principles • Selection of metrics and weights can be challenged by other stakeholders

### Composites for benchmarking

Benchmarking of providers based on only one or a few indicators of quality may be problematic for several reasons. First, benchmarking based upon a few indicators infers a strong correlation of performance across all dimensions of quality, whether measured or not. However, this has not been found in the extant literature. Several articles have highlighted weak correlations among metrics of quality [16,17]. In other words, performance in one aspect of care quality is not necessarily informative about performance in others. It is possible that composite indicators may be better suited to reflect an overall construct of quality.

A second benefit of composite indicators of quality is that they are communicable to diverse stakeholders and may be leveraged to induce competition on quality. Payers of healthcare increasingly employ these measurements to inform and direct patients' choice of providers through selective contracting. Patients may gain from transparent provider competition for quality and through the ability to make informed healthcare choices. While to date there is little evidence that benchmarking information affects patient choice of provider [18], consumer attitudes may change as the quality and dissemination formats of quality information improve. However, any benefit to patients is dependent on the accuracy of classifying providers as superior or inferior. Variation in methods and quality of existing composites may lead to significant misclassification of providers as outliers [19].

Composite indicators are a simplified representation of the underlying quality of care construct. In fact, simplification is their main appeal. There is a danger, however, that overly simplistic policy messages derived from composites may be misleading or misused to support narrow agendas. If the providers being measured perceive the indicators to lack scientific soundness, transparency, or content validity, they are unlikely to produce desired improvements in patient health status. In addition, a summary score may inaccurately suggest that providers are average if good scores on one metric compensate for poor performance on other metrics. In fact, 'average' providers may be 'poor' providers for patients whose needs are within the low scoring performance areas. Some of these dangers can be countered by using dissemination formats that convey results accurately while avoiding oversimplification (such as the ability to 'drill down' into individual components of the composite), and by making the process of indicator development explicit and transparent to all stakeholders. In addition, statistical techniques such as multi-criterion analysis mitigate the problem of performance averaging [15]. Nevertheless, it is likely that composites used for benchmarking will be subject to methodological and political challenge from providers disagreeing with results.

### Composites for quality improvement

Composite indicators might support quality improvement in various ways. They may help providers translate a bewildering wealth of information into action and track effects throughout the care delivery system. To illustrate, the Vermont Oxford Network tracks the quality of healthcare delivery of over 800 neonatal intensive care units worldwide, with clinically rich information available for many processes and care outcomes [20]. It may be difficult for neonatal intensive care providers to translate large volumes of data into effective quality improvement efforts.

A multi-dimensional approach to quality measurement via composite indicators may support such a multi-dimensional approach to quality improvement. Composite indicators and their individual components may identify specific areas for attention, for which specific evidence-based interventions are then developed. The success of improvement can then be cross-checked with the comprehensive measure set to ensure that this focus has not worsened quality of care in another area. However, targeting individual quality metrics may lead to piecemeal rather than system-based efforts in quality improvement. Potentially, larger leaps in improvement may result from systems-based interventions that affect multiple areas of care simultaneously and have the potential to spread [21] throughout the care service and the institution. Improving safety attitudes among staff is an example of a system-based intervention that may improve outcomes and propagate throughout an institution [22]. Whether composites are used to track improvement targeting individual or multiple metrics will depend on local resources, support systems, expertise, and institutional capacity. In either application, composites would allow tracking of overall improvement and their sub-components could alert users to potential concordant or discordant effects of improvement activities on other measures of quality.

Thus, using composite indicators does not imply replacing the measurement of individual metrics of quality. Rather, composites merely summarize the information contained in the individual metrics and make that information more digestible. A synergistic approach of using both composites and individual metrics may permit harnessing the advantages of both.

Recognizing that there are numerous editorial choices in the development of composite indicators, and that quality of care can be defined in overly simplistic ways, we propose a composite-based approach to measuring pediatric care quality by combining the JRC composite development methodology [15] and Profit *et al*.'s quality measurement framework [12].

### Development of composite indicators

As do other organizations, the JRC has significant institutional expertise in developing, applying, and evaluating composite indicators; it has, in fact, published guidelines for composite indicator development [15,23,24]. These guidelines have begun to be used in other settings of healthcare [25]. What differentiates the JRC's approach from that of other organizations is its highly explicit, transparent, and evaluative approach to composite indicator development. Proposed methods promote internal and external statistical and methodological consistency and offer users choices of building blocks at each step in composite indicator construction, tailored to the task at hand.

Table 2 shows the JRC's ten step approach to composite indicators development [15]. We present here a brief summary of this approach along with a theoretical example of composite score development for pediatric intensive care unit (PICU) quality. We refer readers to the JRC handbook [15] for additional detail.

**Table 2:** Developing a composite indicator

Step	Description
**1**	Developing a theoretical framework
**2**	Metric selection
**3**	Initial data analysis
**4**	Imputation of missing data
**5**	Normalization
**6**	Weighting and aggregation
**7**	Uncertainty and sensitivity analysis
**8**	Links to other metrics
**9**	Deconstruction
**10**	Presentation and dissemination

### Example: developing a PICU quality composite indicator

#### Step one: framework

**Figure 1:**
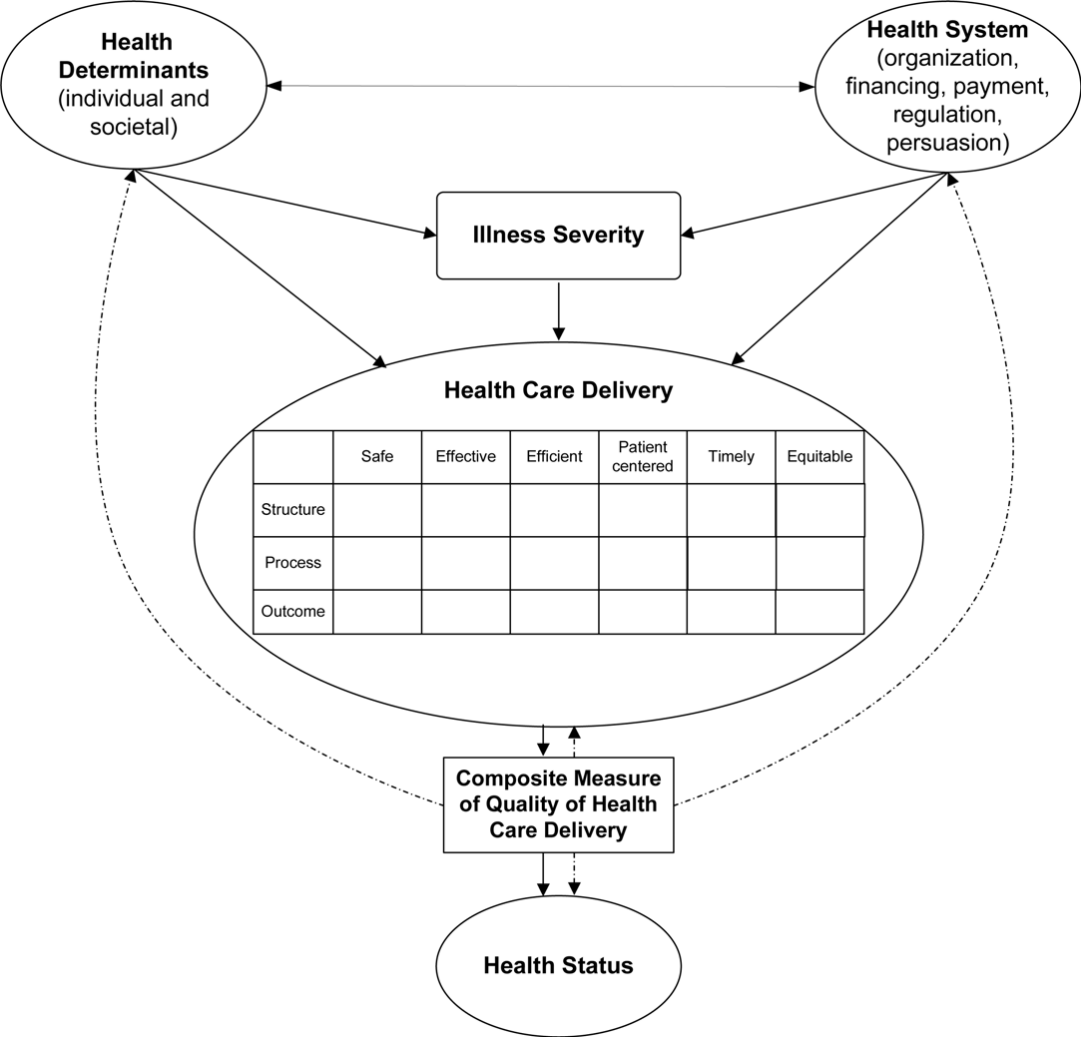
**Theoretical Framework for Measuring Quality of Care**. Solid arrows indicate interactions; dotted arrows indicate potential use of composite indicator to measure healthcare delivery, predict health status and inform health policy at the health systems and societal level. (Adapted from Profit et al. [12]).

We base the framework for a PICU indicator on the work of Arah [26], Roberts [27], the Institute of Medicine (IOM) [28], and Donabedian [29] (see Figure 1). Details of this framework have been described elsewhere [12]. In brief, Figure 1 models a patient's path through the healthcare system and highlights opportunities and challenges for measurement. The model emphasizes innate and external modifiers of health that determine baseline illness severity and that should be addressed via risk adjustment or risk stratification. Quality of healthcare measurement combines the frameworks of the IOM and Donabedian, resulting in a quality matrix (see Table 3). Metrics within the matrix can be combined to form a composite indicator of quality. The resulting composite would combine metrics of structure, process, and outcomes, a combination suggested by others [30], and be based on sub-pillars derived from the IOM domains of quality of care. Metrics within each pillar will correlate among each other and with those of other pillars. Ideally, one would expect moderately high correlations of metrics within pillars and low correlations between pillars. In the end, the composite can serve as an outcome measure, which can then be used to assess the effect of new health policies or changes in medical care on long-term health outcomes.

**Table 3:** Quality matrix for a pediatric intensive care unit quality index

	Safe	Effective	Efficient	Pt-centered	Timely	Equitable
Structure				Nurse-to-patient ratio	Intensivist in house 24 hours a day	

Process	*Medication Safety Practice, Central line infection prevention practice*, VAP prevention practices	*Review of unplanned readmissions*		*Pain assessment on admission, Periodic pain assessment*	Time to receive antibiotics for sepsis	

Outcome	VAP rate, BSI rate, UTI rate, Unplanned extubation rate	*SMR, Unplanned readmission rate*	*Severity adjusted LOS*		Failed extubation rate	

Pt: patient; VAP: ventilator associated pneumonia; BSI: blood stream infection; UTI: urinary tract infection; LOS: length of stay; SMR: standardized mortality ratio.

The italicized items are the eight core metrics in Pedi-QS report. The other items were initially rejected either because of lack of evidence or difficulty in measurement.

Depending on the measurement purpose of the composite, we propose filling the quality matrix with disease- or disease category-specific metrics of quality to create a balanced scorecard of overall quality of care and promote the goal of ensuring that providers are responsive to the quality expectations of all stakeholders, including payers and patients. In many areas of medicine, available metrics may span several domains of quality, may share a cell with other metrics, or may not exist for certain cells of the matrix; the latter measurement state clearly indicates the need for future metric development research. For example, the absence of equity metrics in Table 3's matrix is of note and could be addressed through further research on equity reports [31].

#### Step two: metric selection

Given the high stakes involved with regard to comparative performance measurement, we think that the metric selection process is of cardinal importance to the composite indicator's acceptability among users. Selection should therefore rely on a rigorous and explicit process so that each metric is appropriately vetted with regard to its strengths and weaknesses. Favourable metric characteristics include: importance (*i.e*., relevant domains of care); scientific acceptability, including validity (reflecting the desired measurement construct) and reliability (precision of point estimates); usability (inducing reasonable action plans); timeliness (improving the effect of feedback); and feasibility (data are available and easily retrievable) [32]. In our example, the Pediatric Data Quality Systems (Pedi-QS) Collaborative Measures Workgroup is a joint consensus panel formed by the National Association of Children's Hospitals and Related Institutions, Child Health Corporation of America, and Medical Management Planning tasked with recommending pediatric quality metrics to the Joint Commission[33] In 2005, the Work Group recommended eight process and outcome quality metrics for use in the PICU, which we have placed into the matrix (see Table 3). The selection of metrics may be informed by expert opinion or based on statistical methods. The use of expert opinion and a formal metric vetting process may enhance the composite index' external validity and thus user acceptability. On the other hand, a statistical approach to metric selection may be less time consuming and result in a more parsimonious measure set but may lack external validity with users. Importantly, either approach should result in a measure set that clinically represents the underlying quality construct and balances external validity and parsimony. Future updates of the composite should incorporate user feedback and new scientific evidence, which may require changes to the existing measure set. As mentioned above, metric selection and attribution to domains of care inform the structure of the composite with regard to its sub-pillars. We recommend a minimum of three measures per pillar, meaning that given the dearth of available data, a PICU composite would currently lack at least two domains (e.g., equity and efficiency). Whether a metric, such as severity-adjusted length of stay, can be incorporated into the composite can be investigated by examining whether it statistically maps on another domain.

#### Step three: initial data analysis

In this step, the data are prepared for analysis. Consideration should be given to the exclusion of outlier data points, such that resulting performance ratings are not unduly influenced by extreme values. In addition, the data need to be uniform in their directionality. For example, a high ventilator-associated pneumonia (VAP) rate indicates poor quality, but a high level of compliance with VAP prevention practices indicates the opposite. Thus, in the composite, one of the metrics has to be reverse-coded.

#### Step four: missing data

Treatment of missing data may influence hospital performance assessment. The selected approach to assigning values to missing data should reflect the developers' intent for benchmarking and fair treatment of providers. This requires a fundamental judgement whether data are missing at random or missingness signals differences in the underlying case mix between institutions (*e.g*., missing VAP rate data not randomly distributed but reflecting poor recordkeeping and/or poor outcomes). Missingness status (random versus non-random) can be investigated directly, with a missing data analysis (MDA) establishing whether missingness is associated with measured and available variables of interest. However, these investigations have limits: Variables potentially associated with identified missingness cannot be investigated if they have not been measured within the context of the study at hand and remain external to a MDA, constraining its conclusions. Because many benchmarking activities have reputational and/or financial implications, it may be prudent to assume data are not missing at random. The developer could give providers the benefit of the doubt and assign a probability of zero to missing data, here implying a negative outcome did not occur. However, this may provide an incentive to game the system and not provide data on patients with poor outcomes. A similar incentive is provided if missing data are excluded or imputed using a hospitals' average performance. More sophisticated methods for imputing missing data, based on regression analysis or probabilistic modelling, attempt to impute a true value based on a hospital's results with similar patients [34,35]. Yet even these methods may result in an underestimate if providers intentionally game the system. Conversely, assigning a value of one to a missing data point may punish providers unfairly for something beyond their control, *e.g*., data lost in the abstraction and transmission phase of the benchmarking activity. Nevertheless, this approach may encourage complete record keeping. To be successful, missing value imputation must proceed via a carefully selected strategy appropriate for the dataset under analysis. An inappropriate imputation strategy may itself introduce bias into analytic results. Complete-case-analysis, which sidesteps imputation and missingness by use of missing case deletion (list-wise or pair-wise) will produce biased results when non-random missingness is present. Common imputation strategies, such as mean imputation, last observation carried forward, or mean difference imputation, will also introduce bias into results when missingness is non-random. A multiple imputation strategy, preserving the variance of a variable with missingness, will create multiple imputed values and weights to be combined in producing a consistent outcome estimator while accounting for errors in the imputation process itself [36,37]. Thus, a multiple imputation strategy carefully matched to the characteristics of the dataset containing missingness offers a 'best practice' solution.

#### Step five: normalization

From the selected metrics, a base case composite is constructed using a combination of *a priori *agreed on methods. Metrics with different units and scales cannot be aggregated before being transformed to a common scale (normalization). Of the many existing choices for normalization, ranking and assignment to a categorical scale (*e.g*., star rating) are used most commonly; other choices (*e.g*., standardization; distance to a reference metric) should also be considered and evaluated with regard to their effect on hospital performance. The PICU composite may contain proportions (*i.e*. mortality rate, readmission rate) and continuous metrics (*i.e*. length of stay). These measures have to be normalized (e.g., to ranks or z-scores) to make them compatible for aggregation.

#### Step six: weighting and aggregation

This step is crucial in the development of a composite indicator, because decisions about the attribution of weights to metrics as well as metric aggregation may significantly influence performance assessment results. Weights must reflect the importance, validity, reliability, and malleability of individual metrics; metrics with contradictory quality signals (*e.g*., safe and effective, but not efficient) must be weighted to reflect clinical and policy priorities.

### Weighting

The two basic methods used to arrive at metric weights are statistical (*e.g*., principal component analysis, factor analysis, multivariate techniques) and participatory methods (variations on eliciting expert opinion). Note that equal weighting does not imply an absence of weights: under this approach each metric is given a weight of one. An equal weighting scheme may introduce an element of double counting if two metrics prove to be highly correlated (*e.g*., VAP rates and VAP prevention practices).

Benefits of the statistical approach to weighting include its relative fairness and its freedom from bias. In contrast to the participatory approach, its primary disadvantage is that resultant weights may lack face validity.

Equal weighting has the benefit of simplicity and has been found to result in comparable performance assessment when compared to differential weighting schemes unless differences in weights are very large [38]. This is especially true if the number of metrics included in the composite is large. Because weighting schemes are inherently controversial, they are likely subject to opposition. One approach to addressing such concerns involves the use of data envelopment analysis, which allows each hospital to vary the weights to individual metrics such that the hospital can achieve its optimal position among its peers [39].

### Aggregation

In this phase the metrics are combined to form the composite indicator. The primary decision involved in choosing an aggregation method hinges on whether providers should be allowed to compensate for poor performance in one metric with superior performance in another. There are three principal choices: full compensation (additive), partial compensation (multiplicative), and no compensation (non-compensatory).

Because of its simplicity, the additive aggregation technique is used widely. However, developers need to be cognizant that additive aggregation implies full compensability between metrics and may therefore result in a biased composite indicator, with an error of dimension and direction not easily determined.

Multiplicative aggregation allows for partial compensability, which makes it more difficult to offset a bad indicator with a good one. This is in line with our concept of quality in which a quality performance metric is intended to foster superior quality throughout domains of care and not promote trade-offs between areas of strength and weakness.

Non-compensatory methods, such as multi-criterion analysis, demand achieving excellence in all metrics of quality or at least achieving minimum standards of quality, thereby promoting multi-dimensional improvement efforts. We believe that developers of pediatric composite indicators should seriously consider the use of non-compensatory aggregation methods, so that quality of care in one aspect cannot be traded off another, since negative consequences of poor quality of care in any area of healthcare may have long-term consequences for a child's health and social well being. At the least, we recommend this aggregation method be explored as a variant of indicator construction in uncertainty analysis (see step seven). One variant of non-compensatory methods, the 'all-or-none measurement' approach, has been recently propagated as a means to foster excellence in quality [40]. However, it has been argued that this particular approach is likely imprecise and may provide perverse incentives, such as promoting treatment irrespective of how small the potential benefit and how great the patient burden or risk [41].

#### Step seven: uncertainty analysis

The effect of subjective choices and chance variation in the underlying data on provider performance can be modelled in higher order Monte Carlo experiments. The importance of uncertainty analysis cannot be overemphasized. Composite indicators must be sufficiently robust in discriminating outliers on both extremes of performance in order to enhance their usefulness and engender provider trust. Thus, stability of results in uncertainty analysis provides an important quality check of the composite indicator as well as of the underlying framework and data [42].

#### Step eight: links to other metrics

If composite indicators of quality for related pediatric populations existed, these indicators could be linked to the PICU indicator. Composite indicators, if developed based on compatible methods, can thereby be extended to measure quality at a higher level, such as quality of care at the level of the hospital or the service region in a cross-sectional and longitudinal manner. For example, a composite indicator of quality of related specialties whose patients frequently require PICU care (*e.g*., pulmonology) could be combined with a PICU indicator, and thus provide a better image of quality for specific patient populations across disease episodes. In addition, a PICU indicator can be correlated with indirect measures of quality (e.g., measures of patient safety culture [22]) for purposes of criterion validation of an inherently immeasurable construct.

#### Step nine: deconstruction

For presentation purposes, the composite indicator can be deconstructed to reveal contributions from individual metrics to overall performance. If a measure contributes little to the overall score, the developer may consider removing the variable from the composite for purposes of parsimony. This decision may be moderated by whether or not the measure to be removed is perceived to be of high clinical importance, so that its omission would compromise acceptability of the composite among users. A good example for such an indicator could be mortality. This outcome is generally uncommon and has been shown in the neonatal intensive care setting to be a poor discriminator of overall care quality [43]. Yet, given its clinical importance, most clinicians may prefer its inclusion in a composite.

#### Step ten: presentation and dissemination

Presentation formats can be user-friendly, such as charts that include metrics of uncertainty (*e.g*., confidence intervals). Electronic publications can link to further detail on individual metrics [44].

### Pediatric aspects of composite indicator development

Developing a composite indicator of quality for pediatric care faces several challenges, including paucity of interactions with the healthcare system, paucity of critical health outcomes, and availability of quality of life and prevention metrics. These factors have various implications for measurement that, when taken together, present unique challenges to composite development for pediatric care.

### Paucity of interactions with the healthcare system

The number of yearly admissions for pediatric patients is smaller than that for adults, making sample size a significant issue [45]. Metric development may therefore require ongoing data collection over several years and across multiple institutions. The aggregation of several metrics into a composite indicator may alleviate this problem, in that information from multiple quality metrics can be combined and thereby increase the power to detect a quality signal; however, this is an empirical question and needs to be addressed in future research.

### Paucity of critical health outcomes

As death is an uncommon outcome in children, mortality in isolation is a poor discriminator of care quality [43]. Moreover, mortality does not always represent poor care quality but may reflect appropriate decisions by providers and parents to provide comfort care for children with irreversible and debilitating conditions. Attitudes towards comfort care are likely to vary among providers, regions, and parental caregivers, which further undermines the ability of mortality to discriminate hospital quality of care [46]. Nevertheless, mortality is an important balancing measure, which ensures that hospitals do not receive undue credit for measures that are sensitive to mortality (*e.g*., length of stay). We therefore recommend including mortality in composite indicators measuring the quality of acute care settings. However, its effect on provider performance should be subject to sensitivity analysis, as should be its weighting.

### Quality of life metrics

Health-related quality of life is an important outcome of care quality, but it is difficult to measure in children. Because children under the age of five are typically unable to reliably answer quality of life questions, caregiver proxy assessment has been used as a reasonable substitute [47,48]. However, because parental rating of their children's quality of life may be positively biased [49], health-related quality of life ratings may need to be obtained from health professionals or the general public. Recommendations for cost-effectiveness analysis favour the general public's perspective [50]; yet such ratings are strongly influenced by responder personal experience with health status [51] and may also reflect the availability and quality of chronic care management and the degree of health system integration. In addition, studies by Saigal and colleagues suggest that patient utilities may not be stable over a patient's life, even in light of stable chronic disease [52-55]. This suggests that the effect of patient preferences on provider performance on a composite indicator of quality should be assessed by allowing preferences to vary over a reasonable range in sensitivity analyses. Future research should try to address these important methodological gaps that remain in the measurement of health-related quality of life of young children [56]. Until such research is conducted, the uncertainty in quality of life ratings should be reflected in lower relative weightings, so as to not threaten the external validity of the composite indicator.

### Prevention metrics

Much of the job of pediatric health professionals is to prevent illness or illness exacerbation. Therefore, metrics of primary prevention should be given particular consideration during the metric selection process. Childhood illness may potentially lead to long-lasting, even devastating, adverse outcomes, permanently altering children's developmental trajectories [57]. Thankfully, high quality rehabilitation and educational services can support children's unique adaptation to injury, enabling them to reach full potential [58]. This implies that measurement of healthcare quality should emphasize longitudinal linkages to health outcomes over time, which will provide an opportunity for validation of the composite indicator and offer opportunities for further linkage to additional social well being outcomes to help assess the quality of larger societal systems, including social support and educational systems. Currently, few such metrics exist, and much research will be needed to develop them.

The importance of preventive care services in pediatrics does not necessarily imply that this aspect of care should be attributed higher relative importance compared to measures of acute care in a composite indicator of pediatric healthcare quality. Measure developers will have to make decisions on weighting with regard to the purpose of the indicator, the underlying data, and clinical applicability. For example, measures of preventive care are likely to feature less prominently in a composite of pediatric intensive care than in a composite of ambulatory care. In addition, developers may choose to apply differential weights among preventive care measures based on their value to public health in a given society (*e.g*., the prevention of obesity may be of greater value than administration of polio vaccine).

## Summary

Composite indicators are being more widely used to measure healthcare provider performance and may have benchmarking or quality improvement purposes. However, failure to adopt rigorous indicator development methods will undermine their ultimate usefulness in improving quality and instead encourage physician perception that performance measurement is unreliable and inaccurate [59-61]. Pediatric quality of care measurement presents unique challenges to researchers in this field, and much empirical work remains to create best practice in composite indicator development. However, the combination of JRC's performance metric development methodology with Profit *et al*.'s quality matrix framework may result in a unique approach for quality measurement that is fair, scientifically sound, and promotes the all-important provider buy-in. Future work should evaluate the feasibility and value of the proposed approach in helping make accurate benchmarking and quality improvement decisions.

## Competing interests

The authors declare that they have no competing interests.

## Authors' contributions

JP and LP led the conceptualization, design, writing, and revision of the manuscript. KT contributed to adaption of the content to the pediatric intensive care unit setting. MK contributed to the composition of a revised framework for composite indicator measurement and adaptation of the methods to the healthcare setting. KT, SH, LW, LP, and MK contributed to writing and revision of the manuscript. JP is guarantor of the paper. All authors read and approved the final manuscript.
